# Local ablation of pulmonary malignancies abutting pleura: Evaluation of midterm local efficacy and safety

**DOI:** 10.3389/fonc.2022.976777

**Published:** 2022-08-22

**Authors:** Hong-Tao Hu, Xiao-Hui Zhao, Chen-Yang Guo, Quan-Jun Yao, Xiang Geng, Wen-Bo Zhu, Hong-Le Li, Wei-Jun Fan, Hai-Liang Li

**Affiliations:** ^1^ Department of Minimal-Invasive Intervention, the Affiliated Cancer Hospital of Zhengzhou University & Henan Cancer Hospital, Zhengzhou, China; ^2^ The Affiliated Cancer Hospital of Zhengzhou University & Henan Cancer Hospital, Zhengzhou, China; ^3^ Department of Minimally Invasive Interventional Therapy, Sun Yat-Sen University Cancer Center, Guangzhou, China

**Keywords:** lung malignancies, microwave ablation, radiofrequency ablation, pleura, cryoablation,

## Abstract

**Objective:**

To retrospectively evaluate the efficacy and safety of local ablation treatment for adjacent pleural lung tumors.

**Materials and methods:**

Sixty-two patients who underwent pulmonary nodule ablation at the Affiliated Cancer Hospital of Zhengzhou University were enrolled between January 2016 and December 2020. All patients were followed up with enhanced computed tomography or magnetic resonance imaging within 48 h after treatment and 2, 4, 6, 9, and 12 months after treatment. All patients were followed for at least 12 months.

**Results:**

A total of 84 targeted tumors (62 patients) underwent 94 ablations. In the 12-month follow-up images, 69 of the 84 targeted tumors were completely ablated, 15 had incomplete ablation, and the 12-month incomplete ablation rate was 17.8% (15/84). Of the 15 incompletely ablated tumors, six had partial responses, five had stable disease, and four had progressive disease. The most common adverse event was pneumothorax, with an incidence of 54.8% (34/62). The second most common complication was pleural effusion, with an incidence rate of 41.9% (26/62). The incidence of needle-tract bleeding was 21% (13/62) and all patients were cured using hemostatic drugs. Serious complications were bronchopleural fistula in four patients (6.5%, 4/62) and needle tract metastasis in one patient. Four cases of bronchopleural fistula were found in the early stages and were cured after symptomatic treatment.

**Conclusion:**

Local ablation is effective for the treatment of adjacent pleural lung tumors, and its operation is safe and controllable.

## Introduction

According to statistics from the American Cancer Society, lung cancer is the leading cause of cancer death in the United States, with approximately 350 people dying of lung cancer every day (1). With societal development and the aggravation of air pollution in developing countries, the incidence of lung malignancies is on the rise (2). Surgical resection has always been the main treatment for patients with early non-small cell lung cancer and some patients with pulmonary metastatic diseases (3, 4). However, more than 20% of patients with early non-small cell lung cancer are not suitable for surgery because of various factors (such as old age, severe lung function impairment, or other complications) (3). The standard local treatment option for these inoperable patients is stereotactic body radiotherapy (SBRT), which has a local control rate of 90% for early lung cancer and has achieved an effect similar to that of surgery (5, 6).

In recent years, image-guided local ablation has been rapidly developed as an alternative to radiotherapy, including radiofrequency ablation (RFA), microwave ablation (MWA), and cryoablation. Compared with SBRT, local ablation also has a good local control rate for inoperable early lung cancer (7–9). Many studies have reported the use of ablation in the treatment of early non-small cell lung cancer and oligometastatic lung cancer (10–12), but there are few reports on the ablation of adjacent pleural lung cancer or pulmonary metastases. The development of its efficacy and complications have not been discussed in depth. The purpose of this study was to retrospectively evaluate the local efficacy and safety of local ablation in the treatment of adjacent pleural lung tumors.

## Materials and methods

### Patients and admission criteria

Before conducting this retrospective study, we obtained approval from the ethics committee of the Affiliated Cancer Hospital of Zhengzhou University. All patients in the study received ablation treatment for lung tumors in our hospital and signed a written informed consent form for ablation treatment prior to the treatment. From January 2016 to December 2020, 522 patients with concurrent local ablation of pulmonary malignancies were admitted to our department, including 306 patients who underwent local radical ablation and 62 patients who met the criteria of this study. The inclusion criteria for this study were as follows: (A) the patient’s lung lesion was histologically diagnosed as a malignant tumor; (B) if the lung lesion was a primary tumor, it must be non-small-cell lung cancer; (C) because of personal reasons, the patient was unsuitable for surgical treatment (such as insufficient cardiopulmonary function reserve) or unwilling to undergo surgical resection; (D) the number of lesions in patients was ≤3 and confined to one side of the lung, and the maximum diameter of a single tumor was ≤30 mm; (E) the patient had at least one lesion located ≤5 mm away from the pleura; and (F) if the intrapulmonary lesion was a metastatic tumor, it should be stable for at least 3 months after systemic treatment, with no new metastatic lesion in the lung. The exclusion criteria were as follows: (A) the primary malignant tumor was not effectively controlled; (B) primary lung cancer was accompanied by extrapulmonary metastasis or spread; (C) there were multiple lesions on both sides of the lung; (D) the maximum diameter of a single tumor was >30 mm; (E) imaging indicated mediastinal lymph node metastasis or had invaded the chest wall or mediastinum; and (F) severe coagulation dysfunction, systemic infection, thrombocytopenia, or other conditions unsuitable for lung tumor ablation.

### Pre-treatment examination and planning

All patients underwent comprehensive imaging and laboratory examinations prior to local ablation. This included chest enhanced computed tomography (CT) or magnetic resonance imaging (MRI), as well as complete blood count and coagulation indices in laboratory examinations, including bleeding time, partial thromboplastin time, and international normalized ratio. The patients stopped using anticoagulants or antiplatelet drugs within 3 days to 1 week before surgery, and the coagulation function was rechecked the day before surgery to ensure that it was within the normal range. Before the surgery, two associate professors jointly planned the patient’s position and puncture path according to the location and number of tumors. Prophylactic antibiotics were not routinely administered.

### Ablation procedure

**Figure 1:**
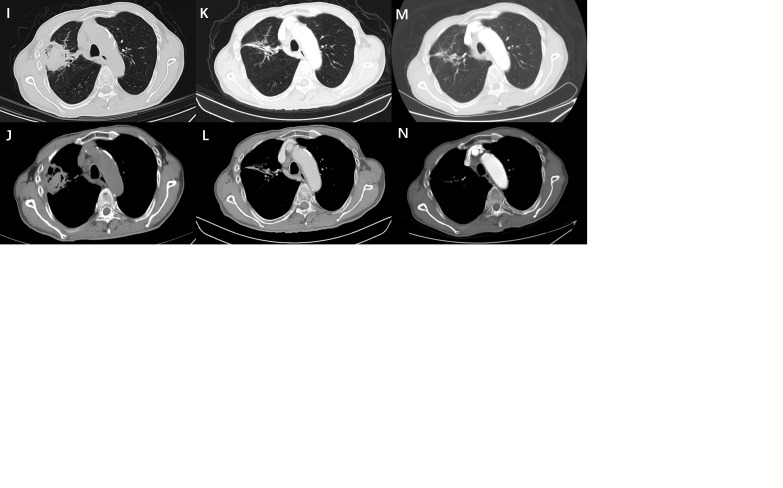
**(A)** A 69-year-old male presented with a tumor in the right upper lobe of his lung. **(B, C)** The biopsy specimen was diagnosed as lung adenocarcinoma. Lung function is poor owing to emphysema and cannot be surgically removed. Radiofrequency ablation was performed at our department. **(D)** After ablation was completed, the computed tomography scan showed a small amount of pneumothorax in the right upper lung. The patient had no symptoms of chest tightness or discomfort and no related treatment was performed. **(E)** The day after the ablation of the lung cancer, the patient complained of fever, chest tightness, and subcutaneous emphysema, and a computed tomography (CT) scan showed increased pneumothorax with subcutaneous emphysema and pleural effusion. **(F)** Considering that the patient had a bronchopleural fistula, a pleural drainage was performed using a 10F pigtail catheter, and anti-infective therapy was given. Ten days after continuous chest drainage combined with anti-infective therapy, the patient’s chest tightness and fever disappeared completely. **(G, H)** A repeat CT scan showed that the pneumothorax and pleural effusion had completely disappeared, and the original lung tumor showed extensive necrosis. The patient recovered and was discharged from the hospital. **(I, J)** The CT scan of the patient 2 months after discharge showed that the extent of tumor ablation was smaller than before, and the contrast-enhanced CT examination 1 year after ablation showed that the necrotic tumor was absorbed, showing a cord shadow **(K, L)**. **(M, N)** Contrast-enhanced CT 2 years after ablation showed that the original residual cable shadow was further absorbed and reduced.

Local ablation was performed by three interventional physicians under sterile conditions in the CT room (HL.L, CY.G, and HT.H, with 20, 20, and 15 years of experience in lung tumor ablation, respectively). Twenty patients were treated under general anesthesia and 42 patients were treated with local anesthesia combined with analgesia. A GE large-hole CT machine was used (the scanning parameters were 30 mAs, 120 kV, and 5 mm thickness), and electrocardiogram (ECG) monitoring was used during the entire process. According to the preoperative planned body position and needle insertion path, the patients were asked to lie on their back, prone, or side on the CT workbench. After scanning and positioning, the needle insertion path was confirmed (**Figure 1A**). After disinfection, a predetermined ablation equipment electrode was used to insert the lesion location according to the designed best path (**Figures 1B, C**). Argon-helium knife ablation was started with all the focal electrodes in place (if there were multiple or large lesions), MWA and RFA was performed successively using a single lesion (if there were multiple lesions), and all tumors on one side of the lung were ablated at one time without serious complications. All ablation procedures were performed in accordance with the recommendations of the equipment manufacturer, and the ablation range was beyond 5 mm on both sides of the tumor to ensure an adequate range of ablation safety. CT scans were used during ablation to assess the adequacy of the ablation range and monitor related complications. If ablation complications occurred, treatment termination was decided according to the severity of the event and the degree of intervention required. Because all tumors in this study were adjacent to the pleura, after treatment, the electrode was removed without any coagulation of the needle track. A CT scan was performed immediately to confirm the ablation range and presence of complications (**Figure 1D**). If any complications occurred, corresponding measures were performed. If there were no complications or treatments, the puncture point was disinfected again and covered with an application. The patients underwent continuous ECG monitoring for 12 h after returning to the ward.

### Follow-up after ablation

A chest CT scan was performed within 48 h after treatment to determine if there were delayed complications, such as delayed pneumothorax (**Figures 1E, F**) and bleeding. CT or MRI follow-up studies were performed at 2, 4, 6, 9, 12, and 18 months after discharge to evaluate efficacy and complications (**Figures 1G–N**). The follow-up CT or MRI scan scheme was plain scanning plus multiphase enhancement. The postoperative CT images were evaluated by two senior doctors (JC.X and QJ.Y, with 20 and 15 years of experience, respectively) who were not involved in the ablation surgery. The shortest follow-up period for patients in this study was 12 months. Complete ablation of the tumor was confirmed by enhancing the enhanced signs of CT or MRI. Irregular focal soft tissue enhancement was considered to be a sign of residue or recurrence. An annular thin-wall symmetric reinforcement of <5 mm was observed 6 months after ablation, which was considered a sign of enhancement of peripheral normal tissue after complete ablation of the tumor (13, 14). A review revealed residual or enlarged focal soft tissue enhancement lesions that were considered as lesions with incomplete primary ablation from 1 to 12 months after the initial ablation (14). The lesions were ablated again, after excluding tumor diffusion or metastasis. The method and process of ablation were the same as those described above.

### Study end point

The main endpoints of this study were single complete ablation rate and complications. The single complete ablation rate was defined as the absence of tumor recurrence within 12 months after the first local ablation. Adverse reactions were evaluated according to the expert consensus for image-guided RFA of pulmonary tumors (2015 version) (14).

### Statistical methods

The Shapiro-Wilk test was used to determine the normality of the quantitative data. Continuous numerical variables of normal distribution are expressed as mean ± standard deviation, and continuous numerical variables that do not conform to normal distribution are described by the median and interquartile range. Count data are expressed as percentages. All statistical analyses were performed using SPSS software (version 23.0; SPSS, Chicago, IL, USA).

## Results

### Patients and tumors

By December 2020, 62 patients were enrolled, including 41 males and 21 females, with a mean age of 59.76 ± 13.94 years. Among the 62 patients with lung tumors, 28 had lung cancer (seven cases of squamous cell carcinoma and 21 cases of adenocarcinoma). There were 34 cases of lung metastasis, including 24 cases of rectal cancer, five cases of liver cancer, and five cases of mammary cancer (**Table 1**). There were 84 lesions were near the pleura (31 subpleural tumors of lung cancer and 53 subpleural tumors of lung metastases). There were 81 lesions in the costopleura and 13 in the diaphragmatic pleura. Among them, 12 lesions were ablated with argon-helium knife (eight patients), 14 lesions with MWA (10 patients), and 58 lesions with RFA (44 patients).

**Table 1:** Baseline characteristics of the study patients.

Characteristics	Number of cases	Number of lesions
	N = 62	N = 84
Gender, No. (%)		
Male	41 (66.13%)	–
Female	21 (33.87%)	–
Age (years), mean ± SD	59.76 ± 13.94	–
Tumor size(mm)*, median (IQR)	19.00(16.00-25.00)	
Primary tumor site, No. (%)		
Lung		
Squamous	7 (11.29%)	8 (9.52%)
Adenocarcinoma	21 (33.87)%	23 (38.10%)
Colorectal	24 (38.71%)	36 (42.86%)
Breast	5 (8.06%)	8 (9.52%)
Live	5 (8.06%)	9 (10.71%)
Treatment		
RFA	44 (70.97%)	58 (69.05%)
MWA	10 (16.13%)	14 (16.67%)
Argon-helium knife	8 (12.90%)	12 (14.29%)

*Multiple lesions in the lung were treated by ablation one by one, and the largest diameter of each lesion was counted here.RFA, radiofrequency ablation; MWA, microwave ablation.

### Treatment and follow-up

The 84 lung lesions that were observed in the 62 patients with malignant lung tumors were successfully ablated. Intraoperative CT showed that all tumors were covered by the ablation range, and the technical success rate was 100%. As of December 31, 2021, all patients were followed up using enhanced CT or MRI after local ablation and completed at least 12 months of follow-up. For patients with suspected tumor edge activity on CT images, enhanced MRI were added to further evaluate tumor activity. During the follow-up period, 54 of 62 patients achieved complete ablation (two patients underwent ablation twice), and 69 of 84 targeted tumors were ablated completely. Even after two ablations (eight patients), 15 target lesions showed tumor residue, recurrence, or progression during the follow-up period. The 12-month follow-up showed that the complete ablation rate of all lesions was 82.1% (69/84) and the 12-month complete response (CR) rate of 62 patients was 87.0% (54/62). Of the eight patients with lung tumor progression after local ablation, six received systemic chemotherapy combined with radiotherapy, and two received systemic chemotherapy. As of the last follow-up, among the 54 patients who achieved complete ablation within 12 months, four patients had new intrapulmonary tumors and three had distant metastasis. Among the eight patients with incomplete ablation, four patients had tumor progression, two were stable, and two died due to multiple organ failure (**Table 2**). Due to the large number of pulmonary metastases cases (54.8%, 34/62) in this study, these patients received other anticancer therapies after disease progression, so the overall survival was not analyzed in this study.

**Table 2:** 12-month complete ablation rates and follow-up.

Follow-up	Complete ablation		Incomplete ablation	
	Number of cases	Number of lesions	Number of cases	Number of lesions
12-month ablation rate	87.10%	82.1%	12.90%	17.86%
Treatment				
RFA	38/44	49/58	6/44	9/58
MWA	9/10	11/14	1/10	3/14
Argon-helium knife	7/8	9/12	1/8	3/12
Treatment after progression				
Systemic chemotherapy	*		2	
Systemic chemotherapy + radiotherapy	*		6	
Condition assessment				
Stable condition	47		2	
Disease progression	7		4	
Death	0		2	

*Since complete ablation was achieved, no other treatments were performed during the follow-up period.

### Complications

No ablation-related deaths occurred during ablation or within 30 days of treatment. The most common complication was pneumothorax, with an incidence of 54.8% (34/62), but only eight patients required fine tube drainage, and no patients needed surgical incision and drainage. Another common complication was pleural effusion formation, with an incidence of 41.9% (26/62), and 16 patients required catheter drainage of pleural effusion. The complications of local ablation included pain, postoperative syndrome, bleeding, coughing, and pleural reactions (**Table 3**). Of the 62 patients treated with local ablation, 20 (31.8%, 20/62) experienced intraoperative or postoperative pain, including 17 cases of mild pain without intervention and three cases of moderate and severe pain requiring drug treatment. However, all symptoms were relieved within 3-5 days after treatment. Postoperative syndromes, including low fever (<38.5°C), nausea, vomiting, and general malaise, occurred in 12 cases (22.7%, 12/62). Coughing was also a common complication in nine cases (19.7%, 9/62), including six mild and three severe cases of coughing. The incidence of needle bleeding was 21% (13/62), and all patients were cured by hemostatic drug infusions. The most serious complications were bronchopleural fistula in four patients (6.5%, 4/62) and needle track implantation in one patient. There were no other serious complications such as thoracic hemorrhage, phrenic nerve injury, or air embolism. Four cases of bronchopleural fistula were found early, and they were cured after intrathoracic intubation and anti-infective treatment (**Figures 1E–H**). One patient was found to have needle tract implantation during the 3-month follow-up and was cured by the second ablation. Two patients had a cavity in the target tumor area on CT 2 months later. During the follow-up period, the cavity was gradually reduced and absorbed in one case, and there was no change in the other case. There were no signs of residual tumor activity or recurrence in these two patients.

**Table 3:** Incidences of complications and associated factors.

Characteristics	Postoperative syndrome	Pain (Mid/moderate to severe)	Cough (Mild to moderate/severe)	Pneumothorax	Pleural effusion	Bleeding	Bronchopleural fistula
Total	12 (19.35%)	20 (17/3)	9 (6/3)	34 (54.8%)	26 (41.9%)	13 (21.0%)	3 (4.8%)
Treatment							
RFA	10	19(16/3)	8 (6/2)	31	25	10	2
MWA	1	0	1 (0/1)	2	0	2	1
Argon-helium knife	1	1 (1/0)	0	1	1	1	0
Primary tumor site							
Lung	4	10 (8/2)	5 (3/2)	16	12	6	1
Colorectal	6	9 (8/1)	4 (3/1)	14	13	5	2
Live	1	1 (1/0)	0	2	1	1	0
Breast	1	0	0	2	0	1	0

## Discussion

Some patients with early lung cancer or oligometastatic lung cancer cannot tolerate surgical resection because of their old age and/or poor cardiopulmonary function. In addition, some patients had previously undergone surgery at other sites and were unwilling to undergo surgical resection again. Therefore, image-guided percutaneous puncture and ablation, such as RFA, MWA, and cryoablation, is an alternative to surgery with the advantages of fewer complications, less trauma, good tolerance, high repeatability, and rapid recovery (15–17).

Although lung tumor ablation has been considered an alternative therapy for unresectable early lung cancer, ablation is still considered high-risk and prone to recurrence for early lung cancer adjacent to the pleura and oligometastatic lung cancer (18). The main reason is that an insufficient safe ablation range can lead to recurrence, and severe pleural injury may lead to bronchopleural fistula (18–19). Second, the ablation treatment for lung tumors is different from that for liver tumors. The lung parenchyma differs from the liver in terms of energy deposition, electrical conductivity, thermal diffusion, and thermal convection (20). The energy required for the treatment of lung tumors is usually lower than that required for the treatment of liver tumors of the same size. Proper control of the ablation range requires further exploration.

Our study showed that the complete ablation rate of tumors at 12 months was 82.1% (69/84), which is lower than the previously reported complete ablation rate of 93% at 18 months (21), and also lower than the 1-year progression-free survival rate of 94.0% reported by Huang et al. (22). The ablation effect may be affected by several factors: a) the tumor is close to the pleura, which limits the operator’s ability to expand the scope of ablation; b) the pain caused by ablation of the adjacent pleural tumor under local anesthesia leads to a lack of sufficient ablation time or course of treatment, and may eventually form a residual tumor. Of the eight patients with incomplete ablation in our study, six were ablated under local anesthesia; and c) there are few choices for the puncture needle path near the pleural tumor, and the puncture technique is difficult, which often requires repeated needle adjustment and is prone to pneumothorax. Once pneumothorax occurs, the distance between the tumor and visceral pleura is further reduced, and the pleura is more likely to be damaged during ablation.

Our results showed that the 12-month CR rate in this group was 87%, which is similar to the previously reported 18-month CR rate of 88% (21). This may be due to the high proportion of primary lung cancer in our study (45.2%, 28/62) and only 15% of primary lung cancers, as reported by de Baere et al. (9/60) (22). Thomas et al. (23) reported a complete ablation rate of 73.1% for lung metastatic cancer ablation. The ablation success rate of patients with diameter ≤3 cm was higher than that of patients with diameter >3 cm. In addition, their study indicated that there was no significant interdependency between the histopathological types of metastatic tumors and the ablation effect. Our study also showed that the complete ablation rate of tumors was unrelated to the histopathological type of metastatic tumors. The complete ablation rate of metastatic lung cancer in our study was higher than that previously reported, which may be related to the fact that all tumors in our study were <30 mm, and the number of cases was lower.

Previous studies have shown that the duration of surgery and ablation of the free edge of the tumor are significantly related to the size of the tumor, and the ablation edge beyond the edge of the tumor by at least 5 mm is a key factor in ensuring complete ablation (24, 25). Our study demonstrated that for lung tumors adjacent to the pleura, complete ablation was achieved in most tumors, even if the margin of the tumor was <5 mm away from the pleura. We admit that this is a high rate of complete ablation combined with a high incidence of complications. The high success rate may be due to the following reasons: a) advances in equipment and technology have made the scope of ablation more accurate, ensuring the scope of ablation while minimizing damage to the surrounding tissue; b) multiple intermittent ablations were performed with low power and long time mode, and CT scanning was performed at each ablation interval to determine the ablation range in time to achieve accurate control of the ablation range; and c) for lesions <10 mm, pleural damage can be minimized by puncturing the needle parallel to the pleura adjacent to the tumor and close to the medial edge of the tumor. According to our experience, avoiding vertical pleural puncture of the tumor for ablation can reduce pleural damage. Nevertheless, there were four cases of bronchopleural fistula in this study, with an incidence rate of 6.5%, which was much higher than the 0.2%-0.6% reported in literature (26, 27). This is directly related to the fact that the distance from the lung tumor to the pleura was ≤5 mm in all patients in this study. Although the above methods have been adopted for treatment, the occurrence of bronchopleural fistula cannot be avoided, which shows that the degree of pleural injury caused by ablation is the most important factor affecting this complication.

Although the incidence of bronchopleural fistula is very low, it is difficult to manage, and its long-term presence may lead to more serious complications, such as infection and intractable pneumothorax. This can be life-threatening, and its treatment is challenging (28). Needle ablation has been reported to be prone to bronchopleural fistula (29); however, needle ablation was not performed in our study. Therefore, pleural injury caused by the ablation procedure is the main cause of bronchopleural fistula formation. Rapid cure of bronchopleural fistula was achieved in four cases in our study. Our team benefited from early diagnosis, early treatment, and localized bronchiolar injury. It has been reported that the presence of bronchopleural fistula should be highly suspected for the persistence of delayed or recurrent pneumothorax because even a large number of pneumothorax cases will disappear quickly after drainage (26, 29–30). In our study, four patients showed no significant improvement after continuous postoperative drainage of pneumothorax and developed pleural effusion. We immediately administered anti-infective treatment and 8F-10F drainage tube placement to strengthen drainage. After 2 weeks, the pneumothorax and pleural effusion disappeared, and the patient was cured. The treatment of tracheopleural fistula in previous reports is very difficult, most of which require multiple surgical methods to perform the intervention (31). The rapid recovery of these four patients by minimally invasive surgery may be related to minor pleural damage because of the small subpleural nodules, which are terminal bronchioles. All these factors promote tracheopleural fistula closure after adequate anti-infective treatment and drainage. However, bronchopleural fistula after ablation of the central tumor may not be closed easily by drainage and anti-infective treatment alone, which requires early surgical intervention. On the other hand, previous studies have shown no significant difference in technical success, technical efficacy, local tumor control, and complications with artificial pneumothorax compared with no artificial pneumothorax (32). Although studies have shown that artificial air/hydrothorax can reduce pain in patients (33), it does not reduce the occurrence of bronchopleural fistula. Therefore, early detection, diagnosis, and treatment are effective treatments for this serious complication.

In this study, three ablation methods were used, mainly RFA, but there was no significant difference between the complete ablation rate, serious complications, and ablation equipment, indicating that as long as the ablation equipment is used correctly, RFA, MWA, or cryoablation can safely complete the ablation of lung tumors near the pleura. In this study, there was a case of needle track implantation caused by poor respiratory coordination under local anesthesia and repeated adjustment of the needle path. For this situation, we believe that there are two ways to reduce needle track implantation. (A) General anesthesia and apnea techniques can be used to improve puncture accuracy, and (B) needle track implantation metastasis could be avoided by sending it into the ablation electrode through the coaxial needle external sheath after coaxial needle puncture to the tumor edge. Further studies are needed to confirm the feasibility of this method.

This study had some limitations. First, the retrospective nature of this study may lead to data bias. Second, the small sample size cannot fully demonstrate the effectiveness of treatment and the incidence of complications. Third, this study used a variety of ablation methods, and the choice of different ablation methods is mainly related to doctors’ treatment preferences, patients’ choices, and medical insurance policies, which may also lead to biased results. These deficiencies are expected to be addressed through larger sample sizes and multicenter prospective studies in the future.

In short, good results can be achieved by using appropriate ablation methods and techniques for lung tumors near the subpleura, which are not suitable for surgical treatment. Although combined with high complication of tracheopleural fistula, if diagnosed early and if correct treatment is provided, this serious complication can be treated effectively in a short time and related death can be avoided. In the future, the rate of serious complications could decrease owing to the development of technology and equipment.

## Data availability statement

The raw data supporting the conclusions of this article will be made available by the authors, without undue reservation.

## Ethics statement

The studies involving human participants were reviewed and approved by Ethics Committee of the Affiliated Cancer Hospital of Zhengzhou University. The patients/participants provided their written informed consent to participate in this study. Written informed consent was obtained from the individual(s) for the publication of any potentially identifiable images or data included in this article.

## Author contributions

Conception and design: Hai-LL. Patient selection and treatment: H-TH, X-HZ, C-YG, Q-JY, XG, W-BZ. Data collection, analysis and interpretation: H-TH, X-HZ, C-YG, Q-JY, XG. Data interpretation: Hai-LL, H-TH, C-YG, Q-JY. Undertook steering committee activities and critical statistical processing: H-TH, X-HZ. Manuscript writing: Hai-LL, H-TH, X-HZ. Manuscript reviewing: Hai-LL, H-TH,W-JF, Hong-LL. All authors contributed to the article and approved the submitted version.

## Funding

Technology Major Project of the Ministry of Science and Technology of China (2018ZX10303502). Henan Province Medical Science and Technology Research Project (201701032). Medical Education Research Project of Henan Province (Wjlx2021334). Henan Province Natural Science Foundation (212300410403). Henan Province Natural Science Foundation (222300420574).

## Acknowledgments

We thank all our authors listed in this manuscript, and also thank all the patients participated in the study.

## Conflict of interest

The authors declare that the research was conducted in the absence of any commercial or financial relationships that could be construed as a potential conflict of interest.

## Publisher’s note

All claims expressed in this article are solely those of the authors and do not necessarily represent those of their affiliated organizations, or those of the publisher, the editors and the reviewers. Any product that may be evaluated in this article, or claim that may be made by its manufacturer, is not guaranteed or endorsed by the publisher.
